# Is There a Role of Inferior Frontal Cortex in Motor Timing? A Study of Paced Finger Tapping in Patients with Non-Fluent Aphasia

**DOI:** 10.3390/neurosci4030020

**Published:** 2023-09-18

**Authors:** Chrysanthi Andronoglou, George Konstantakopoulos, Christina Simoudi, Dimitrios Kasselimis, Ioannis Evdokimidis, Evangelos Tsoukas, Dimitrios Tsolakopoulos, Georgia Angelopoulou, Constantin Potagas

**Affiliations:** 1 Neuropsychology and Language Disorders Unit, First Department of Neurology, Eginition Hospital, National and Kapodistrian University of Athens, 115 28 Athens, Greececpotagas@otenet.gr (C.P.); 2 First Department of Psychiatry, Eginition Hospital, National and Kapodistrian University of Athens, 115 28 Athens, Greece; 3 Research Department of Clinical, Education and Health Psychology, University College London, London WC1E 6JB, UK; 4 Multisensory and Temporal Processing Laboratory (MultiTimeLab), Department of Psychology, Panteion University of Social and Political Sciences, 136 Syngrou Ave., 176 71 Athens, Greece; 5 Department of Psychology, Panteion University of Social and Political Sciences, 176 71 Athens, Greece

**Keywords:** aphasia, Broca’s area, motor timing, finger tapping

## Abstract

The aim of the present study was to investigate the deficits in timing reproduction in individuals with non-fluent aphasia after a left hemisphere lesion including the inferior frontal gyrus, in which Broca’s region is traditionally localised. Eighteen stroke patients with non-fluent aphasia and twenty-two healthy controls were recruited. We used a finger-tapping Test, which consisted of the synchronisation and the continuation phase with three fixed intervals (450 ms, 650 ms and 850 ms). Participants firstly had to tap simultaneously with the device’s auditory stimuli (clips) (synchronisation phase) and then continue their tapping in the same pace when the stimuli were absent (continuation phase). Patients with aphasia demonstrated less accuracy and greater variability during reproduction in both phases, compared to healthy participants. More specifically, in the continuation phase, individuals with aphasia reproduced longer intervals than the targets, whereas healthy participants displayed accelerated responses. Moreover, patients’ timing variability was greater in the absence of the auditory stimuli. This could possibly be attributed to deficient mental representation of intervals and not experiencing motor difficulties (due to left hemisphere stroke), as the two groups did not differ in tapping reproduction with either hand. Given that previous findings suggest a potential link between the IFG, timing and working memory, we argue that patients’ extra-linguistic cognitive impairments should be accounted for, as possible contributing factors to timing disturbances.

## 1. Introduction

Time processing is of fundamental importance for human behaviour. In an ever-changing, dynamic environment, actions like dancing, driving or speaking are characterised by certain speed, sequence of events and time intervals. Timing and rhythmic relations are also core aspects of normal speech. With regard to the sense of time, diverse cortical regions have been suggested to be engaged in different timing processes [1]. According to the fMRI literature, explicit timing (e.g., repetitive tapping) typically recruit the basal ganglia and supplementary motor area, whereas in implicit timing (e.g., drawing movements), activation of the prefrontal and parietal cortex and the cerebellum [2,3,4]. The brain networks underlying the perception of sub-second and supra-second durations are dissociable as well, with sub-second intervals being associated mainly with cerebellum and supra-second intervals being linked to prefrontal cortex and premotor area [1,5,6]. In particular, it has been proposed the operation of a cognitive timing mechanism linked to memory and attention during the performance of longer time durations, whereas an automatic mechanism seems to be responsible for stimuli in the range of milliseconds [7].

Besides the involvement of the aforementioned brain regions in temporal processing in healthy individuals, evidence from previous studies in persons with language disorders has focused on the participation of Broca’s region in timing. For example, the speech of patients with non-fluent aphasia is disrupted by long pauses and irregular intervals between syllables and words. Moreover, linguistic studies have shown that impaired temporal processing may underlie the pathology of syntactic processing [8], phonological form [9] and prosody [10] in patients with non-fluent aphasia due to a left-lateralised prefrontal lesion, indicating a possible role of Broca’s region (i.e., posterior portion of the inferior frontal gyrus), among other cortical and subcortical regions in temporal processing that is not limited to speech production.

A growing body of neuroimaging evidence indicates that Broca’s region, in addition to its linguistic functions, such as syntactic comprehension [11], lexical identification, grammatical inflection and phonological processing [12], appears to be engaged in action execution and observation, music execution and listening, and [13,14] learning and preparing motor sequences [15], as well. Interestingly, sequencing and timing are common aspects of all these functions. Therefore, it has been suggested that Broca’s region is multifunctional, adapted to the regulation of sequential activity in several different domains [14,16] and that the left hemisphere dominance for language comes from the specialization of the left hemisphere for rapid temporal processing and integration [17,18]. Findings from neuroimaging studies of timing in healthy individuals offer support to this assumption. Broca’s region was found to be activated by the perception of sequential sounds in PET studies [19], while in the study of [20] using fMRI, the left posterior, superior portion of Broca’s region was activated during the temporal manipulation of linguistic and non-linguistic strings (syllables and hummed notes, respectively). Moreover, bilateral or unilateral activation of the interior frontal gyrus (IFG)has been associated with temporal processing in fMRI studies [21,22,23,24]). In addition, evidence from magnetoencephalography is provided by [25], who showed that bilateral activation of the IFG, as well as bilateral premotor regions and occipital areas, was specifically associated with timing regarding stimuli with duration < 0.5 s, whereas the involvement of the right IFG and parietal regions were associated with attentional and working memory aspects of the timing paradigm, such as sustaining the representation and comparing the tones, especially those with longer durations (i.e., 1100 ms).

Previous studies in patients with aphasia have reported deficits in temporal processing that might be related to lesions in Broca’s region, such as the perception of rapid temporal patterns in speech sounds [26,27,28,29] and in non-speech auditory or visual stimuli [27,30,31,32]. Recent evidence has shown that patients with non-fluent aphasia demonstrate poor temporal [33] and rhythm processing ability [34], as well, suggesting the possible role of central auditory processing in these performances and speech fluency, in general. In another study, left-brain-damaged patients with dysphasia were severely impaired in rhythm perception and production, implying the left hemisphere’s specialisation in temporal processing and rhythm [35], which was also confirmed in a more recent study in which aphasic patients were evaluated in the reproduction of rhythmic patterns [36]. On the contrary, no difference has been found between left hemisphere stroke patients with and without aphasia in temporal processing involving longer intervals, 2–12 s (often known as “cognitive” timing) [37].

According to the aforementioned assumption on the multifunctional role of Broca’s region one could expect that lesions in posterior part of the inferior frontal gyrus might cause a general timing deficit that can affect not only linguistic behaviour, but each kind of motor output [15,38]. Therefore, comparing individuals with and without lesion in this area on a time-based task in which stimuli and responses are independent of speech might provide clearer evidence on a possible general role of the IFG in temporal processing. In the present study, we sought to examine temporal processing in patients with non-fluent aphasia using a task that assesses timing not related to speech, i.e., the paced finger-tapping task. The finger-tapping test is a neuropsychological tool for evaluating motor function and upper extremities coordination in both neurotypical controls and clinical population [39]. It can be either externally generated by utilizing pacing stimuli that will be reproduced by participants or internally generated, when they reproduce tapping in the absence of stimuli [40].

In the paced finger-tapping task participants are asked to first tap in time with computer-generated tones separated by a fixed inter-tone interval (synchronisation phase). After a series of tone-paced responses, the tones are discontinued, and participants are required to continue tapping at the established pace (continuation phase). An fMRI study of healthy subjects during pace finger tapping with their right hand found increased activation of the right IFG (BA44) in the continuation phase of the task, indicating that this region is part of the network involved in the internal generation of precisely timed movements [41]. However, in a more recent study, Pretto and colleagues [42] using EEG found strong activation of the left IFG during synchronisation with a metronome beat in healthy, young participants.

In the present study, we hypothesised that patients with non-fluent aphasia after a lesion affecting the IFG would present poorer timing performance than healthy individuals in both the synchronisation and continuation phases of the task. Greater motor-timing deficits were expected for patients during the continuation phase of the task because in this phase, tapping is related to mental representations of time in the absence of auditory stimuli. We also predicted that patients’ timing deficits would be more pronounced in the faster tempos of the task, as it has been reported by previous researchers assessing temporal perception in speech i.e., [29] and non-speech stimuli i.e., [30] in patients with aphasia.

## 2. Methods

### 2.1. Participants

The clinical sample was recruited from the Neuropsychology and Language Disorders Unit of the 1st Neurological Department of the Eginition Hospital in Athens. Aphasia diagnosis was established with the short form of the Boston Diagnostic Aphasia Examination (BDAE), adapted in Greek [43]. Healthy participants were recruited from local communities (Attika and Korinthos counties). Inclusion criteria for the control subjects were no history of neurological or psychiatric disease, head injury or alcohol/drug abuse. All participants were native Greek speakers and all patients had relatively preserved language comprehension. All participants could make use of their right and left hands and had normal hearing acuity. Thirty-eight of the participants were consistent right-handers, one of the patients was a consistent left-hander and another one was ambidextrous. Patients’ neuroimaging data (based on CT and/or MRI) have been carefully examined by an experienced neuroradiologist, blind to the scope of current study. All patients had a brain lesion from a single ischemic stroke that included posterior part of inferior frontal gyrus. All participants had been informed about the research procedures and given written informed consent as approved by the Ethics Committee of Eginition Hospital.

### 2.2. Task Procedure

The paced finger-tapping task was performed using a response box that consisted of a round, metallic, haptic sensor on a rectangular, wooden surface and was connected with an electric apparatus which produces auditory pacing stimuli, i.e., inter-tone intervals (clips) and a red light as a signal for the onset of the test. The haptic sensor has to be touched with the index finger and was connected to a personal computer (PC) in which the MATLAB programme was installed so as to record the participants’ timing performance. A pair of headphones connected to the PC was also used.

Three inter-tone intervals of 450 ms, 650 ms, and 850 ms were selected for this research. In each trial of the synchronisation phase, a series of 13 clips of only one of the aforementioned intervals was presented. The order of the inter-tone intervals presentation was randomly selected by the MATLAB programme so as to control for learning effects. Each hand was tested three times in each of the three tempos (inter-tone intervals 450 ms, 650 ms and 850 ms). Each synchronisation phase was followed by a continuation phase that allows for 13 finger taps in the absence of the auditory pacing stimuli, as in the study by [44]. Means and standard deviations (SD) of the differences of inter-tap intervals (ITIs) from the tempo (inter-tone intervals) were recorded for each trial of the synchronisation and continuation phases.

Participants were tested on an individual basis in a quiet room. The duration of the experiment was approximately 40 min per participant. Both oral and written instructions for the finger tapping task were provided to the participants. All participants had to take a trial run in order to reassure they had clearly understood all the instructions and to familiarise themselves with the task. In case of cancellation of a trial, the participants had the opportunity to try again up to 6 times.

### 2.3. Statistical Analysis

An unbalanced, mixed design was implemented. Four factors were included. The first factor was “Group” with two levels: (i) individuals with aphasia and (ii) individuals without aphasia. This was a non-true and fixed, between-subjects factor. The second factor was “Phase” with two levels: (i) synchronisation phase and (ii) continuation phase. This was a true and fixed, within-subjects factor. The third factor was “Hand” with two levels: (i) right hand and (ii) left hand. This was a non-true and fixed, within-subjects factor. The fourth factor was “Tempo” with three levels: (i) 450 ms interval, (ii) 650 ms interval, and iii) 850 ms interval. This was a true and fixed, within-subjects factor.

Two dependent variables of time processing were measured in continuous scale: (a) accuracy, i.e., mean of the differences of ITIs from the inter-tone intervals, and (b) variability, i.e., SD of the differences of ITIs from the inter-tone intervals. Therefore, this was a quasi-experimental, without pre-measurement, 2 × 2 × 3 three-way MANOVA with repeated measures on three factors.

Repeated-measures MANOVA was carried out using the IBM SPSS Statistics Version 20 software. Descriptive analysis of the data and one-way ANOVA were performed prior to the MANOVA. The correlation assumption for the dependent variables, discrepancy and accuracy, was tested with the Pearson statistical criterion [45]. The sphericity assumption for the repeated measures was examined via the Mauchly’s W criterion. A MANCOVA with the same design and education, age, gender and preferred hand as covariates was conducted to examine the effect of potential confounding factors.

## 3. Results

Eighteen individuals with non-fluent aphasia (14 male) with a mean age of 52.6 years and 22 healthy participants (12 male) with a mean age of 40.23 years were recruited for the present study. The mean of the years of education was 14.3 for the patient group and 15.8 for healthy participants.

### 3.1. Descriptive Analysis of Finger Tapping Data

**Figure 1:**
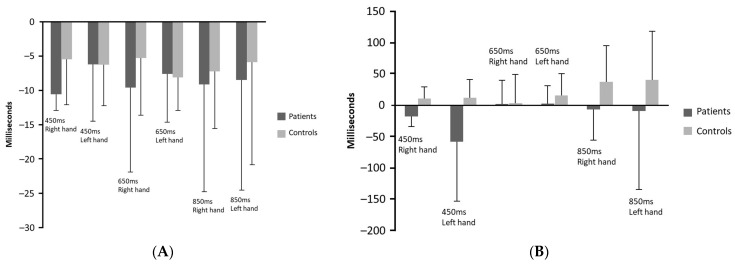
Accuracy in 3 tempos (450, 650, 850) between patients and control groups in both phases. (**A**) accuracy–synchronisation phase; (**B**) accuracy–continuation phase.

**Figure 2:**
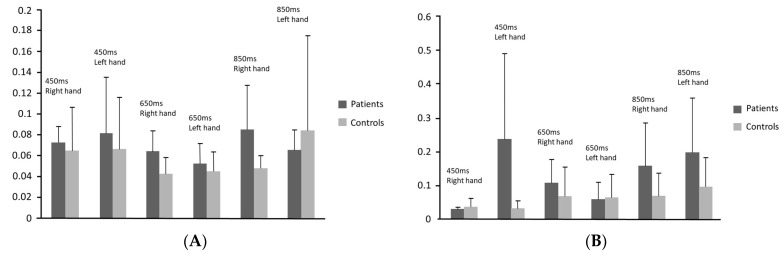
Variability in 3 tempos (450, 650, 850) between patients and control groups in synchronisation and continuation phases. (**A**) Variability–synchronisation phase; (**B**) variability–continuation phase.

Means and the standard deviations of accuracy and variability of the three actual tempos reproduced by the participants with and without non-fluent aphasia with either hand in the synchronisation and the continuation phases are illustrated in Figure 1 and Figure 2, respectively. As shown in Figure 1, the most extreme discrepancy from the tempos was produced by the patients in the continuation phase for the 450 ms inter-tone intervals with left hand (getting faster). The patients produced the larger negative discrepancy (getting slower) in the continuation phase for the 650 ms inter-tone intervals with left hand. Figure 2 shows that the greater variability of responses was observed in the patients’ group during the continuation phase for the 450 ms inter-tone interval.

### 3.2. One-Way ANOVA

An exploratory, one-way ANOVA testing the between-subjects effects revealed statistically significant differences between the patient group and healthy participants in the accuracy and the variability of timing performance, as shown in Table 1. The homogeneity of variance assumption was tested with Levene’s criterion and was confirmed for 14 out of the 24 conditions. Bonferroni pairwise comparisons revealed that the patients presented a significantly increased negative discrepancy (delay) from the stimulus tempo compared to control group. Moreover, patients showed a significantly greater variability in tempos reproduction than healthy participants.

### 3.3. Repeated Measures MANOVA

The correlation analysis revealed 39 significant Pearson coefficients in the 66 pairs of the dependent variables of accuracy and variability, confirming the correlation criterion for MANOVA. The Mauchly’s W sphericity assumption for repeated measures was confirmed for the variability of the tempos reproduced [χ^2^(2) = 0.93, *p* = 0.927]. The assumption was further confirmed for the accuracy in the interaction of the variables hand and tempo [χ^2^(2) = 0.87, *p* = 0.085) and for the variability in the interaction of the variables phase and tempo [χ^2^(2) = 0.99, *p* = 0.898]. Finally, the sphericity assumption was confirmed for both the accuracy and the variability in the interaction of the variables hand, phase and tempo [χ^2^(2) = 0.880, *p* = 0.095 and χ^2^(2) = 0.906, *p* = 0.160, respectively].

Based on the aforementioned results, a 2 × 2 × 3 MANOVA with repeated measures was carried out to test the differences between the patient and the control group in the accuracy and the variability of timing performance. As shown in Table 2, significant main effects of hand (right/left), phase (synchronisation/continuation) and tempo (inter-tone intervals 450 ms/650 ms/850 ms) were found. There also were significant interactions of the group (with/without aphasia) with phase and tempo but not with hand, regarding the variability of tapping performance. In terms of accuracy, the only significant interaction was between the effects of group and tempo.

Analysis of these interactions within groups with pairwise Bonferroni comparisons revealed the following:

(a) Differences between right and left hand were detected only in patients who showed increased variability in inter-tone intervals reproduction with the right hand.
(b) No significant differences between the synchronisation and the continuation phase were detected in accuracy of tapping performance of the patient group. However, significantly greater variability of the tempo reproduced was detected in patients in the continuation phase. Healthy controls had significant difference in accuracy between the two phases (delayed responses to the stimuli tempo in the synchronisation phase versus accelerated responses in the continuation phase), whereas no significant differences between the synchronisation and continuation phases were found in variability of tapping performance.

The pairwise comparisons between patient and control groups revealed the following significant differences:

(a) The variability of tapping performance was greater in patients in both the synchronisation [F(5, 34) = 10.07, *p* < 0.001] and the continuation phase [F(5, 34) = 8.78, *p* < 0.001].
(b) Patients had delayed responses compared to the control group particularly in the continuation phase [F(5, 34) = 5.23, *p* = 0.001]. It is noteworthy that in this phase, the healthy participants reproduced accelerated responses in comparison to the stimuli inter-tone intervals.
(c) More precisely, patients delayed significantly compared to healthy participants when tapping with the left hand in the 450 ms tempo in the continuation phase and when tapping with the right hand in 450 ms tempo in both phases and in 850 ms tempo in the continuation phase. Patients had significantly greater variability of performance compared to control group when tapping with the right hand in 650 ms inter-tone interval in the synchronisation phase and in the 850 ms inter-tone interval in both phases as well as when tapping with the left hand in 450 ms in both phases and in 850 ms inter-tone interval in the continuation phase.

Finally, a MANCOVA including the years of education, the age, the gender and the handedness as covariates revealed no significant effects of these factors on the timing performance of the participants with and without non-fluent aphasia.

## 4. Discussion

The findings of the present study supported our hypothesis that patients with non-fluent aphasia due to a lesion on the left hemisphere, including the inferior frontal gyrus, would present timing deficits measured by the paced finger-tapping task. Significant differences between stroke patients with non-fluent aphasia and healthy participants were found in time performance in the synchronisation and the continuation phase of the task. In particular, patients with non-fluent aphasia demonstrated:

1. Greater variability and poorer accuracy than healthy individuals in overall tapping performance;
2. Greater variability of their responses in both synchronisation and continuation phases compared to healthy participants;
3. Lagged tapping rates only in the continuation phase compared to healthy participants (patients reproduced slower tapping rates, whereas healthy participants faster ITIs than the stimuli tempo);
4. Significantly poorer performance in terms of variability in the continuation than the synchronisation phase, whereas there was no significant difference in healthy participants’ performance between the two phases of the task;
5. No significant difference in the accuracy between the two phases of the task, whereas healthy participants had significantly accelerated responses in the continuation phase.

Taken all together, these results seem to be in agreement with our hypothesis that patients’ impairments would be greatest for the phase requiring mental representation of time. In the continuation phase, the variability of patients’ responses was significantly greater than during the synchronisation phase and their tapping rates were significantly slower. This is in line with the findings of a previous study, in which individuals with aphasia reproduced, with less accuracy and greater variability, simple rhythmic patterns by memory (continuation phase) [36]. In contrast to the patient group, healthy participants accelerated their tapping rates in this phase. Similar findings regarding accelerated tapping performance in healthy population has been also reported by previous studies, suggesting that individuals tend to demonstrate negative constant errors (i.e., reproduction of shorter ITIs compared to the fixed tempi) due to the lack of feedback information of the auditory stimuli in the continuation phase [46,47]. The same performance pattern has been also attributed to the transition from synchronization to continuation phase of the tapping task, leading participants to reproduce ITIs that are less than 50% of the fixed interval [48], whereas other researchers have associated this acceleration to age-related motor processes [49]. 

Patients’ difficulties in reproducing the tempo in the absence of the auditory stimulus suggest impairments in temporal processing. In line with this, in the study by [50] wherein patients with non-fluent aphasia were asked to mentally group sequences of identical acoustic stimuli and create an individual rhythmic pattern, they acquired a new strategy relying on mental counting rather than on automatic temporal integration.

Our results are in line with previous studies that used time discrimination, time estimation or temporal-order judgement tasks with speech sounds [26,27,28,29] or non-speech stimuli [27,30,31,32] and found timing deficits in non-fluent aphasia. Our study is the first to examine time processing in non-fluent aphasia using the paced finger-tapping task with isochronous tempos indicating time deficits in a coordinated motor activity. Finger tapping has been used to study temporal processing in a variety of motor disorders, as the single repetitive movement of tapping greatly minimises processing demands imposed by spatial and quantitative aspects of motor control [51]. However, motor demands could be a confounding factor in the present study as all participants with aphasia had suffered stroke in the left hemisphere that may result in movement difficulties in the right hand. Contrary to this assumption, there was no significant difference between patients and control group in measures of overall tapping performance with either hand (i.e., no significant interaction between “hand” and “group” effects in the MANOVA). Differences between the two groups were found regarding performance with either left or right hand in certain tempos. If movement difficulties accounted for patients’ poorer tapping performance significant differences between the two groups should be found specifically for the right hand. Within the patient group performance was poorer with the right than the left hand in terms of variability but not regarding accuracy. However, one would expect more delayed responses with the right hand, i.e., poorer accuracy, due to our patients’ movement difficulties. Moreover, since significant differences between the two study groups were found in both accuracy and variability with either left or right hand, patients’ poorer tapping performances do not seem to be secondary to movement difficulties.

The cognitive demands of the finger-tapping task might be another confounding factor in our study. Rhythmic and temporal motor tasks require the contribution of important cognitive functions, especially attention and working memory. During the synchronisation phase in the task, attention plays a central role, whereas in the continuation phase, the reproduction of the inter-tone intervals by memory is required. Since patients exhibited poorer tapping performance mainly in the latter phase, the role of possible working memory deficits is of crucial importance. A substantial amount of the literature has established that the role of working memory, i.e., the ability to temporarily store and process information in real time, is central to both higher-level cognitive processing as well as to language processing and that individuals with aphasia demonstrate working memory deficits affecting many aspects of linguistic and non-linguistic processing [52,53]. On the other hand, recent findings in patients with aphasia suggest a possible primary deficit in information retention rather than impairment in working memory per se [54]. These findings are in agreement with the assumption made by [41] about the role of the IFG within the neural network underlying temporal processing during finger tapping, i.e., a subsystem consisted of the IFG and the superior temporal gyrus (STG) that mediates the retention of an internal auditory representation of the target interval duration. At any rate, the potential relationships between working memory and time processing deficits in non-fluent aphasia warrant further investigation.

Contrary to our initial hypothesis, in the present study, patients performed more poorly than healthy participants not only in faster but also in slower tempos of the finger-tapping task. However, the ITIs used in our study were within the range of duration in which temporal deficits has been found in patients with non-fluent aphasia in previous studies examining discrimination of temporal sequencing [30] or duration [27] of auditory stimuli, and temporal-order between two successive acoustic events [32]. We did not investigate temporal processing involving longer intervals, i.e., >2 s, in which the left IFG may be not involved [37]. Our findings suggest that non-fluent aphasia may be related to deficits in temporal information processing specifically regarding mental representation of rapid temporal patterns.

Our results are in agreement with the notions that the IFG is a multifunctional brain region [13,14,15] and part of multiple neural networks including the network that underlying temporal processing, i.e., time perception and motor timing [55]. Our patients had suffered lesions including Broca’s region in the left IFG and exhibited impairment in the continuation phase of finger tapping, whereas in a previous fMRI study in healthy participants activation of the right IFG was specifically associated with the continuation phase of the task [41]. Gelfand & Bookheimer [20] have suggested the role of the left Broca’s region during the performance of temporal strings, while other fMRI studies using tasks that involved temporal patterns (rhythms) or timed sequences found increased bilateral activation of IFG associated with the perception and the reproduction of the rhythm [22,55,56,57]. In PET studies using rhythmic music tasks, activation of the left IFG has been associated with the perception of rhythm [19], while activation of the right IFG seems to contribute specifically in more cognitive and abstract representations of the rhythm, such as differing periodicities [58]. Neuroimaging studies on estimation or discrimination of duration revealed contradictory findings regarding the laterality of IFG activation, possibly due to differences in tasks and measures used and in other aspects of study design [23,24,25,59,60]. Finally, a recent meta-analysis of the connectivity findings on Broca’s region from functional neuroimaging studies identified a cluster within the region (posterior-ventral cluster) specifically associated with rhythmic sequencing, while other clusters were related to speech and cognitive functions [61]. Hence, we expect that future research on the role of the IFG would clarify multiple functions of this region, at least partially lateralised, that contribute to temporal information processing.

## 5. Conclusions

The present research represents the first study to date that has used the paced finger-tapping task with isochronous tempos to study timing in non-fluent aphasia and more specifically, in patients with brain lesions on the left hemisphere that includes the inferior frontal gyrus. Our study revealed that the patient group had motor timing difficulties independent of speech and not related to motor difficulties. Our findings provide further evidence in favor of the hypothesis that the IFG region is multifunctional and indicate its possible involvement in time processing more generally. Disorders of time perception and generation in patients with non-fluent aphasia are worthy of further investigation. Future neuroimaging studies investigating finger-tapping in aphasia, in relation to the possible association between timing performance and working memory, might shed light on the role of the posterior part of the IFG region in time processing. Moreover, knowing the role of the IFG in temporal reproduction might be of value in the rehabilitation of people with non-fluent aphasia [62,63].

## 6. Limitations

A weakness of the present study was the lack of patients’ digital neuroimaging data, which could give us the opportunity to better illustrate the association between their timing impairments and the exact location of brain lesions. Future research in a larger sample of patients with post-stroke aphasia and the employment of both sub- and supra-second durations could shed light on the type of patients’ timing disturbances. Moreover, the recruitment of patients with non-fluent aphasia with lesions, including not only IFG but also other brain regions, could disentangle the neuroanatomical background of timing processing deficits in these patients.

## Figures and Tables

**Table 1:** One-way ANOVA testing the differences between patients with non-fluent aphasia and healthy participants in the accuracy and the variability of the produced rhythm in finger tapping.

Effect	Measure	df	F	*p*
Group	Accuracy	1.38	6.886	0.012
	Variability	1.38	9.684	0.004

**Table 2:** Significant main effects and interactions in a 2 × 2 × 3 MANOVA with repeated measures comparing patients with non-fluent aphasia with healthy participants for the accuracy and the variability in the synchronisation and the continuation phases of finger tapping with the right and the left hand in the three different tempos.

Effect	Wlik’s Λ	Measure	df	F	*p*
Hand	0.801	Variability	1.38	7.320	0.010
Phase	0.438	Accuracy	1.38	8.623	0.006
		Variability	1.38	13.338	0.001
Tempo	0.431	Accuracy	2.37	6.864	0.002
		Variability	2.37	14.523	<0.001
Phase × Group	0.749	Variability	1.38	12.260	0.001
Tempo × Group	0.670	Accuracy	2.76	4.517	0.014
		Variability	2.76	5.238	0.007
Hand × Phase	0.812	Variability	1.38	6.885	0.012
Hand × Phase × Group	0.763	Variability	1.38	11.398	0.002
Hand × Tempo	0.581	Variability	2.76	8.154	0.001
Hand × Tempo × Group	0.625	Variability	2.76	10.234	<0.001
Phase × Tempo	0.368	Variability	2.76	7.913	0.001
Phase × Tempo × Group	0.631	Variability	2.76	8.478	<0.001
Hand × Phase × Tempo	0.624	Variability	2.76	9.282	<0.001
Hand × Phase × Tempo × Group	0.513	Variability	2.76	8.944	<0.001

## Data Availability

Data are available upon reasonable request to the corresponding author.
